# The Emerging Role of Robotic Surgery among Minimally Invasive Surgical Approaches in the Treatment of Hypopharyngeal Carcinoma: Systematic Review and Meta-Analysis

**DOI:** 10.3390/jcm8020256

**Published:** 2019-02-18

**Authors:** Armando De Virgilio, Oreste Iocca, Luca Malvezzi, Pasquale Di Maio, Raul Pellini, Fabio Ferreli, Giovanni Cugini, Giovanni Colombo, Giuseppe Spriano

**Affiliations:** 1 Otorhinolaryngology Unit, Humanitas University, Humanitas Clinical and Research Center-IRCCS, 20089 Rozzano, Italy; oi243@nyu.edu (O.I.); luca.malvezzi@humanitas.it (L.M.); fabio.ferreli@humanitas.it (F.F.); giovanni.cugini@humanitas.it (G.C.); giovanni.colombo@humanitas.it (G.C.); giuseppe.spriano@hunimed.eu (G.S.); 2 Otorhinolaryngology Unit, Department of Surgical ad Biomedical Sciences, University of Perugia, Piazza dell’Università, 1, 06123 Perugia, Italy; dimaio.p86@alice.it; 3 Department of Otolaryngology-Head and Neck Surgery, Regina Elena National Cancer Institute, 00144 Rome, Italy; pelliniraul@yahoo.it

**Keywords:** hypopharynx, robotic surgery, transoral surgery, TORS, pharynx

## Abstract

The aim of this systematic review with meta-analysis was to investigate the available literature on transoral approaches in the treatment of hypopharyngeal squamous cell carcinoma, with a special focus on transoral robotic surgery (TORS). A systematic review was conducted according to the PRISMA (preferred reporting items for systematic reviews and meta-analyses) check-list, and 15 studies were included. Five of the included studies evaluated TORS, while ten studies focused on transoral laser microsurgery (TLM) for the treatment of early or advanced stage hypopharyngeal cancer. Overall, survival rates of TLM and TORS studies, analyzed together in the cumulative meta-analysis, were 66.4% (95% confidence interval (CI) 54.3%–76.7%) at 36+ months of follow up. The TORS subgroup showed a higher cumulative survival rate (85.5%, 95% CI 55.8%–96.5%) compared to TLM (58.5%, 95% CI 46.6%–69.6%). Cumulative data showed that 29.3% (95% CI 24.0%–35.3%) of deaths were attributable to cancer. The results were similar between TLM and TORS studies. The larynx function preservation cumulative rate was 94.3% (95% CI 91.8%–96.1%). The results were similar among the two subgroups. The present review supports the use of transoral approaches in the treatment of hypopharyngeal cancer. TORS is oncologically sound and provides excellent functional results with low complication rates.

## 1. Introduction

Squamous cell carcinoma of the hypopharynx (SCCHP) constitutes 3% to 7% of head and neck cancers [1,2] and despite advancements in surgery and chemoradiotherapy treatment options, its prognosis remains poor. Due to the lack of symptoms in the early phase of the disease, the majority of hypopharyngeal cancers present at the advanced stage. This has important repercussions on survival rates, which greatly vary according to tumor stage. The reported disease-specific survival rate for early-stage disease is around 46% [3], while for later stages, it is less than 30% [4].

There is no agreement on the best treatment approach for hypopharyngeal cancer. Definitive concurrent chemoradiotherapy (CCRT) with the aim of organ preservation has been developed in the last two decades with acceptable rates of success. However, avoiding surgery is not a guarantee of functional preservation, given the early and late side effects of chemotherapy and radiation. On the other hand, traditional open surgery approaches are associated with substantial morbidity. For this reason, it has been an ongoing debate as to which treatment options are best compared to others.

Transoral approaches allow access to hypopharynx and tumor removal without the complications associated with open surgery. In detail, transoral laser microsurgery (TLM) was introduced by Strong and Jako [5] and later developed by Steiner [6]. TLM can minimize the sacrifice of healthy tissue and for this reason it can be considered as an alternative to non-surgical cures. Transoral robotic surgery (TORS) applications in otorhinolaryngology were developed by Weinstein and O’Malley in 2005 [7]. Initial indications for TORS involved base of tongue neoplasms, but later, the applications expanded to the hypopharynx, parapharyngeal space, and supraglottic larynx.

These minimally invasive options changed the perspectives on treatment options for patients affected by tumors of the hypopharynx, mostly because surgical treatment can spare the patients the early and late complications associated with chemoradiotherapy.

The aim of this systematic review was to investigate the available literature on transoral approaches to hypopharynx squamous cell carcinomas with a special focus on TORS, analyzing survival, functional results, and complication rates.

## 2. Materials and Methods

A systematic review was conducted according to the PRISMA (preferred reporting items for systematic reviews and meta-analyses) check-list. We searched PubMed, Embase, and Scopus up to August 1, 2018 for studies evaluating transoral approaches to hypopharynx squamous cell carcinoma. Regarding inclusion criteria, all types of studies examining hypopharynx cancer were searched to provide summary estimates on survival and complications. Only English language studies were considered. When duplicate studies were identified, the one with the most recent or the most complete data was included. Study authors were contacted if incomplete or unclear information was reported. Cumulative reports in the form of single-arm meta-analysis were reported regarding cumulative overall survival, cumulative proportion of deaths attributable to cancer, and cumulative rate of larynx preservation. The choice of the outcome cumulative deaths attributable to cancer, instead of disease specific survival (DSS), was dictated by the fact that this specific outcome was scarcely or not uniformly reported in many of the included studies. On the other hand, the crude number of deaths attributable to cancer was widely described in most of the papers. For this reason, it was considered a usable and informative outcome in a single arm meta-analysis like this one. Studies with less than 10 patients were excluded from the statistical analysis.

Logit proportion transformation of the data was performed for the analysis on overall rates with a 95% confidence interval (CI). The DerSimonian–Laird method was the chosen method for the random effects meta-analyses. The *I*^2^ statistic was used to assess heterogeneity. All the analyses were performed using the R software for statistical computing (R 2.10.1; “meta” package).

### Data Extraction

Two investigators (A.D.V., O.I.) searched for studies independently, and the identification of studies was performed through screening of the titles and selecting the abstracts for full-text inclusion.

The reviewers screened all the abstracts for inclusion and finally analyzed the full texts of the included articles. Any author disagreement was resolved by a third author (G.S.). The characteristics of the included studies are summarized in a synthesis table.

## 3. Results

**Figure 1:**
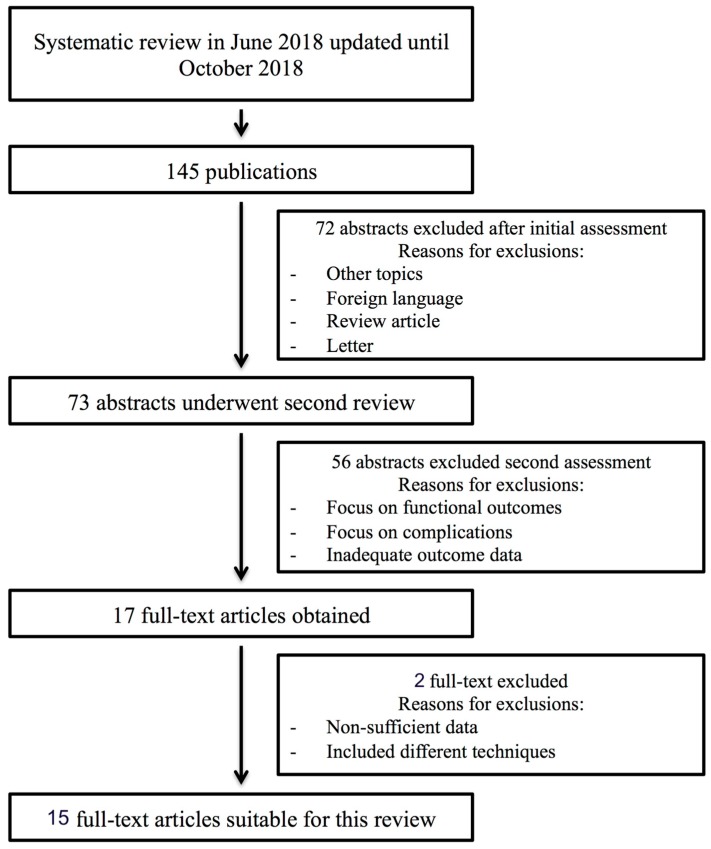
Flow chart for the inclusion of studies.

One-hundred and forty-five studies were identified through a database search (Figure 1). A total of 73 studies were screened for abstract evaluation. Of these, 17 were reviewed for full text, and 15 were finally included in the systematic review [8,9,10,11,12,13,14,15,16,17,18,19,20,21,22] (Table 1). All the included studies were retrospective or prospective observational studies.

Five of the included studies evaluated TORS [8,9,12,14,15], while ten studies focused on TLM [10,11,13,16,17,18,19,20,21,22] for the treatment of early or advanced stage hypopharyngeal cancer.

All of the studies provided estimates of survival, either Overall Survival (OS), Disease Specific Survival (DSS), or Disease-Free Survival (DFS). Locoregional Control (LC) percentages were reported in 12/15 studies. Description of functional outcomes was provided when reported in the included studies.

In all of the reports, the mean patient population age was above 50 years. The male:female ratio was 11:1.

TORS studies were based on a limited number of patients, the maximum being the study of Mazerolle et al. [8] with 57 cases, while some TLM case series had more than 100 cases.

In all studies, the need for adjuvant therapy was decided based on international guidelines with regard to the histological final results. Indications for adjuvant radiotherapy, with or without chemotherapy, were based on the usual criteria for a poor prognosis, comprising positive margins on surgical samples, perineural or lymphovascular invasion, the involvement of more than one lymph node, and extracapsular nodal spread.

### 3.1. TORS Studies

Mazerolle et al. [8] focused on pyriform sinus carcinoma. They performed TORS treatment on 57 patients, where 98% of patients were T1–T2. The overall survival rate was 84% at two years and 66% at four years. The disease free survival rate was 74% at two years and 50% at four years. Four patients needed enteral alimentation via gastric tube. Two tracheotomies were needed in patients undergoing radiotherapy, which were subsequently removed.

Park et al. [9] evaluated the long-term oncological and functional outcomes in patients affected by all stages of SCCHP in a time span of six years. Ten out of 38 patients underwent neoadjuvant chemotherapy prior to TORS and ipsilateral elective neck dissection (level II to IV) in cN0 patients. At the final follow up, 17 patients were alive with no evidence of disease (44.7%). Just seven patients died from cancer-related deaths, the others from other causes. Regarding functional outcomes, 76.3% of patients showed a favorable swallowing ability, and just one patient became permanently dependent on tube feeding. Three patients required permanent tracheostomy. The authors concluded that TORS and simultaneous neck dissection with or without adjuvant therapy is comparable to conventional therapies and allows for more rapid functional recovery. Data from all 38 cases were included in the statistical analysis.

Wang et al. [12] focused on early T SCCHP and followed up ten patients for three years after surgery. Four patients received RT as adjuvant treatment, the others received surgery alone. All patients showed excellent results at three years with no local recurrence or loss of the voice box.

Durmus et al. [14] reported a case series of five patients treated with robotic surgery alone or robotic surgery plus CO_2_ laser. Two patients were T2/N2 or T3 and received adjuvant CCRT, while the others received no adjuvant treatments. Only one patient required temporary tracheostomy and parenteral gastrostomy. All patients then returned to their normal activities without swallowing or speech problems into the successive three months after surgery.

Lorincz et al. [15] followed up with five T1–T2 SCCHP patients who were treated with TORS surgery. Two of them underwent adjuvant treatment via RT or CCRT. Only the patients treated with CCRT had severe aspiration at three months, and this was lost at six months follow up. All of the others experienced no penetration or mild aspiration that resolved at six months.

Durmus et al. [14] and Lorinz et al. [15] studies were excluded from the cumulative survival/functional analisis because they included less than 10 cases. They were considered for descriptive purposes.

### 3.2. TLM Studies

Weiss et al. [10] examined 211 patients treated with TLM for hypopharyngeal cancer. Five-year estimates for local control were 88%, 74.8%, 77.3%, and 61.8%, respectively for pT1-4a tumors. The OS was 68.2% for stage I–II, 65.9% for stage III, 44.5% for stage IVa. A nasogastric feeding tube was placed in 75% of patients with a mean duration of 11 days. Four percent of patients required a permanent feeding tube. Post-operative bleeding occurred in 22% of patients. Tracheotomy was necessary in eight cases. Comparable results were obtained in similar case series by Tomifuji et al. [16], Vilaseca et al. [21], and Rudert et al. [22].

Breda et al. [11] evaluated 37 patients treated with transoral laser microsurgery for hypopharyngeal cancer from stage II to stage IV. Five-year overall survival rates were 90% for stage 63.5% for stage II and 39.8% for stage IVa. Five-year control rates were 90% for stage II and 87.5% for stage IVa.

Canis et al. [13] evaluated the oncologic results of 13 patients who underwent treatment for hypopharyngeal squamous cell carcinoma. Five-year local control was 78.9% and DSS was 71.8%. In terms of post-operative complications, two patients experienced post-operative bleeding, and nine required feeding with nasal feeding tubes with a mean duration of 15 days.

Kuo et al. [17] performed a comparison between open pharyngolaryngectomy and endoscopic laser microsurgery approaches. The last group was composed of 25 patients, of which 22 were classified as T2–T3. Overall Survival was 67% at five years, and disease specific survival was equal to 76%. A nasogastric tube was necessary in 22 patients, and tracheotomy was performed in 14 patients. Comparable results were obtained by Leong et al. [19] in 11 patients, of which 76% were alive at the last clinic review. One of the surviving patients needed a permanent percutaneous gastrostomy.

Karatzanis et al. [18] achieved similar results in 119 patients with early T hypopharyngeal cancers. For T1 cases, the DSS was 78% and for T2, it was 70% with 34 deaths attributable to disease. Major reported complications were postoperative bleeding, aspiration, fistula, and granulation tissue formation. Permanent tracheotomy was necessary in 2.5% of patients. Also, the necessity of permanent gastrostomies was recorded to be 2.5% of the total.

Martin et al. [20] reported five-year local control rates in 172 patients of 84% for T1, 70% for T2, 75% for T3, and 57% for T4a. Five-year Overall Survival was 72% for stages I–II, 61% for stage III, and 43% for stage IVa. There were 42 deaths attributable to cancer.

### 3.3. Cumulative Results

**Figure 2:**
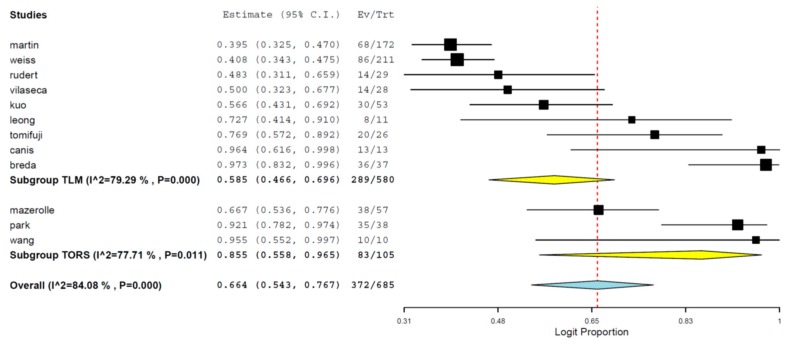
Subgroup meta-analysis including transoral robotic surgery (TORS) and transoral laser microsurgery (TLM) groups regarding overall Ssurvival estimates (95% confidence interval (CI)). Ev, events; Trt, treatment.

Overall survival rates of TLM and TORS studies analyzed together through the cumulative analysis were 66.4% (95% CI 54.3%–76.7%) at 36+ months of follow up (Figure 2). The TORS subgroup showed a higher cumulative survival rate of 85.5% (95% CI 55.8%–96.5%) compared to TLM (58.5%, 95% CI 46.6%–69.6%), although the TORS studies included a higher proportion of patients with lower stage disease compared to TLM. The results were collected including all T or stages of T, because the majority of studies did not include clear stratification of data. Overall, results were heterogenous among studies (*I*^2^ 84.08%, *p* < 0.05, Figure 2).

**Figure 3:**
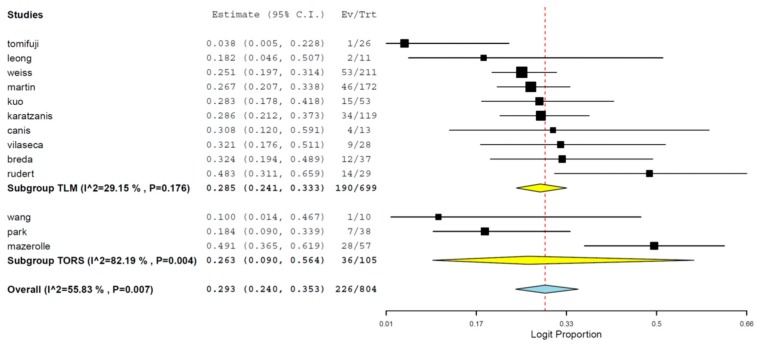
Subgroup meta-analysis including TORS and TLM groups regarding cumulative deaths attributable to cancer estimates (95% CI).

Cumulative data showed that 29.3% (95% CI 24.0%–35.3%) of deaths were attributable to cancer. The results were similar between TLM and TORS studies. Heterogeneity was not significant for this outcome (Figure 3).

**Figure 4:**
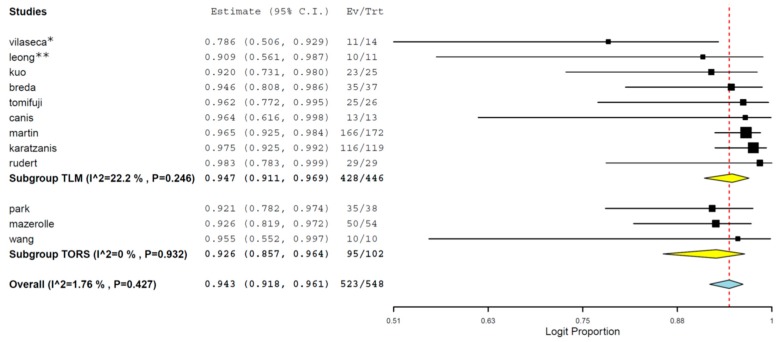
Subgroup meta-analysis including TORS and TLM groups regarding swallowing and voice Function preservation estimates (95% CI). * Only patients without recurrence were evaluated for this outcome **. One patient was not treated with curative intent.

The larynx function preservation cumulative rate was 94.3% (95% CI 91.8%–96.1%). The results were similar among the two subgroups (Figure 4). Heterogeneity was not significant.

## 4. Discussion

The prognosis of hypopharyngeal cancer is the worst among head and neck cancers, and there has been no significant change in its survival rate for decades. Furthermore, there remains no consensus on optimal upfront treatment: surgery with adjuvant radio/chemotherapy or radio/chemotherapy alone.

Radical open surgery could require sacrifice of the larynx and pharyngeal sensory nerve plexus, causing the loss of functions, such as phonation and swallowing. The loss of these functions plus the mutilating effect provided by total laryngectomy are associated with a significant reduction in patient quality of life. Thus, with the development of concurrent chemoradiation, much research has been done towards increasing organ preservation rates and improving quality of life using chemoradiation. However, according to recent studies [23], the combined use of chemotherapy and radiation increases toxicity and could lead to chronic injury and fibrosis in the pharyngeal mucosa, worsening swallowing function.

The literature includes no definitive results in terms of survival benefit when comparing surgery vs. chemoradiation. Axon et al. [24] examined 143 patients with post-cricoid carcinoma and found a significant difference favoring a surgical approach in terms of five-year OS for patients treated with surgery plus RT versus RT alone. On the other hand, Hall et al. [25], in 2009, performed a population-based comparison, on 595 patients, between surgery with adjuvant RT versus definitive RT and identified no statistically significant difference between the two groups. Prospective studies have been conducted comparing various surgical and non-surgical treatment options, with conflicting results. Beauvillain et al. [26] studied 92 patients affected by hypopharyngeal cancer and treated with induction chemotherapy with cisplatin followed by surgery and radiotherapy or radiotherapy alone. The five-year survival rate was 37% in the surgery group compared to 19% for the radiotherapy group, five-year local control rates were 63% versus 39% respectively.

Tsou et al. [27] retrospectively analyzed 202 patients with hypopharyngeal carcinoma (HPC) who were treated with either surgery plus concurrent chemoradiotherapy or concurrent chemoradiotherapy alone. The five-year disease-specific survival rate was 80% for stage I–II, 44.8% for stage III, and 14.3% for stage IV disease. Surgery plus concomitant chemoradiotherapy led to a better survival rate than CCRT plus salvage surgery in patients with stage III–IV HPC.

Unclear conclusions were reported in the retrospective analysis by Harris et al. [28] performed on 166 patients, in which just a trend was found in favor of surgery plus RT or CCRT versus non-surgical approaches alone.

In the absence of a preferred strategy, treatments evolved towards less invasive approaches, and over the decades, more refined surgical techniques have been developed, such as TLM and, more recently, TORS. Among TLM approaches, recently, videoendoscopic techniques have been described, for example, the transoral videoendoscopic approach by Tomifuji et al. [16] and the endoscopic laryngopharyngeal surgery by Kawasaki et al. [29]. These approaches may replace the use of the conventional microscope and classical microlaryngeal instruments with videoendoscopic tools.

In general, transoral approaches maintain the main advantages of the open approaches compared to CCRT: specimens of primary lesion and neck dissection provide important pathologic data [30,31]. Based on this pathologic information, patients can be classified into high or low-risk groups, modulating the eventual administration of adjuvant radio/chemotherapy. For example, adjuvant therapy in early cases should be indicated for high-risk groups with findings of positive margin, perineural or vascular embolism, and extracapsular spread. On the other hand, radiotherapy can be saved in low-risk groups without poor prognostic factors and can be used to treat recurrent diseases or secondary primary malignancies in the future. Decreased radiation doses, and thus reduced global cumulative toxicity, is related to better functional results and minimal complication rates [30,31].

The results of the present review confirm that survival rates of hypopharyngeal cancers treated with a transoral approach with or without adjuvant therapy, either TORS or TLM, may be comparable to the ones of patients treated via open surgery and non-surgical approaches. The overall survival rate of TLM and TORS studies analyzed together was 66.4% (95% CI 54.3%–76.7%) at 36+ months of follow up. Cumulative data showed that 29.3% (95% CI 24%–35.3%) of the deaths were attributable to cancer.

In general, transoral procedures provide lower morbidity and complication rates than open surgery because of the limited need for tracheotomy, the preservation of the suprahyoid muscles and nervous plexus (and therefore normal swallowing), more rapid postoperative recovery of phonation, avoidance of reconstruction, and decreased hospital stay [20].

These considerations are largely supported by our review in which we have reported excellent functional results. The larynx function preservation cumulative rate was 94.3% (95% CI 91.8%–96.1%).

The low morbidity and complication rates could prevent delays in adjuvant treatment such as radiation or chemotherapy. Because the operation time for transoral approaches is shorter than for open surgery, TORS and TLM might be safer for elderly patients in poor general condition or with other medical diseases.

The aim of the present study was not to compare one treatment with the other. Instead, the scope of the review was to highlight the outcomes of the transoral surgical approaches in the management of hypoharyngeal carcinoma. In fact, heterogeneous results emerged: TORS studies included a higher proportion of patients with lower stage disease (e.g., Mazerolle et al. [8]) compared to TLM (e.g., Steiner et al. [6]). Furthermore, the study by Park et al. [9] even considered advanced cases, included 10 patients treated by neoadjuvant chemotherapy. For these reasons, a clear comparison between the two transoral approaches is not possible. For descriptive purposes, we report that the TORS subgroup showed a cumulative survival rate of 85.5% (95% CI 55.8%–96.5%), while that of TLM was 58.5% (95% CI 46.6%–69.6%). The cumulative data showed that 29.3% (95% CI 24.0%–35.3%) deaths were attributable to cancer, and the results were similar between TLM and TORS studies. The larynx preservation cumulative rate was 94.3% (95% CI 91.8–96.1%). The results were similar among the two subgroups.

## 5. Conclusions

Transoral surgical approaches are nowadays proposed as alternative treatments for patients with hypopharyngeal cancer in selected cases. According to literature data, TLM and TORS provide excellent functional results [32,33,34]. Despite reporting good survival and local control, the majority of studies have short follow-up periods and do not report anatomic inclusion/exclusion criteria. For these reasons, further controlled studies are needed to demonstrate the oncological value of transoral surgery in the treatment of hypopharyngeal carcinoma.

## Figures and Tables

**Table 1:** Summary of demographics, survival, and complications of the included studies.

Series	Technique	No. Pts	M/F	Age	Early/Advanced	OS 3 years	OS 5 years	DSS 3 years	DSS 5 years	LC 3 years	LC 5 years	DFS 3 years	Complications
Mazerolle et al. 2018 [8]	TORS	57	52/5	60	56/1	84%	66%						5% (3) bleeding; 2% (1) pharyngeal fistula; 2% (1) neck hematoma
Park et al. 2017 [9]	TORS	38		66.7	22/16			100% I–II	100% I–II	97%	100% I–II	100% early	5% (2) bleeding; 8% (3) aspiration pneumonia
								74% III–IV	74% III–IV		97%	68.6% advanced	
Weiss et al. 2017 [10]	TLM	211	189/22	57.4	32/179	81.5% I–II	68.2% I–II	96.7% I–II	96.7% I–II		88.1% T1		10.4% (22) bleeding; 0.9% (2) pharyngeal fistula
						79% III	65.9% III	86% III	83.8% III		74.8% T2		
						54% IV	44.5% IV	71% IV	62% IV		77.3% T3		
											61.8% T4		
Breda et al. 2017 [11]	TLM	37	37/0	58.7	12/25	80.3% I–II	63.5% I–II	85.3% I–II	74.1% I–II		100% T1		2.7 aspiration pneumonia; 8.1% bleeding
						57.1% III	39.5% III–IV	85.7% III			87.4%T2		
						53.1 IV	39.8 IV	59% IV			100%T3		
											50%T4		
Wang et al. 2016 [12]	TORS	10	10/0	60	6/4	100% I–II	100% I–II	100% I–II	100% I–II	100% I–II	100% T1	100% I–II	0
						50% III–IV	50% III–IV	100% III–IV	100% III–IV	100% III–IV	100% T2	100% III–IV	
Canis et al. 2015 [13]	TLM	13					47.9%		71.8%	78.9%	78.9%	48.5%	
Durmus et al. 2015 [14]	TORS	5	4/1	59.8	3/2								0
Lorincz et al. 2015 [15]	TORS	5	4/1	63.4	4/1	100% I–II	100% I–II	100% I–II	100% I–II	100% I–II	100% T1	100% I–II	0
						0% III–IV	0% III–IV	100% III–IV	100% III–IV	100% III–IV	100% T2		
Tomifuji et al. 2014 [16]	TOVS	26			13/13		78% all		96% all		90% all		NOS
Kuo et al. 2013 [17]	TLM	25	24/1	58	9/16	79% all	67% all	83% all	76%all		92% all		13% (3) aspiration pneumonia; 4% (1) subcutaneous emphysema; 4% (1) local infection
Karatzanis et al. 2010 [18]	TLM	119	107/12	55.4	47/72				84.4% I	90% T1	90% T1		5% (6) bleeding; 4% (5) aspiration; 1% (1) fistula
									77.1% II	88% T2	83.1% T2		
									68.2% III		85.4% all		
Leong et al. 2010 [19]	TLM	12			2/9	71%		71%				71%	
Martin et al. 2008 [20]	TLM	172	153/19	57									
Vilaseca et al. 2004 [21]	TLM	28	27/1	56.6	6/22			100% I–II	100% I–II	100%	100% T1		10.7% (3) aspiration pneumonia; 7.1% (2) bleeding; 3.5% (1) pharyngeal fistula
								45.2% III–IV	45.2% III–IV	91.6%	91.6% T2		
										56.2%	56.2% T3		
										100%	100% T4		
Rudert et al. 2003 [22]	TLM	29	24/5	53.1	9/20	62%	48%	70%	58%		87.5% T1	74%	3.4% (1) bleeding
											63.2% T2		
											100%T3		
											100% T4		

No. Pts, Patients; M, male; F, female; OS 3y, overall survival 3 years; OS 5y, overall survival 5 years; DSS 3y, disease specific survival 3 years; LC 3y, locoregional control 3 years; LC 5y, locoregional control 5 years; DFS 3y, disease free survival 3 years; TORS, transoral robotic surgery; TLM, transoral laser microsurgery; TOVS, trans oral video surgery.
